# Expansion of Human Limbal Epithelial Stem/Progenitor Cells Using Different Human Sera: A Multivariate Statistical Analysis

**DOI:** 10.3390/ijms21176132

**Published:** 2020-08-25

**Authors:** Raquel Hernáez-Moya, Sheyla González, Arantza Urkaregi, Jose Ignacio Pijoan, Sophie X. Deng, Noelia Andollo

**Affiliations:** 1 Department of Cell Biology and Histology, School of Medicine and Nursing, Biocruces Bizkaia Health Research Institute, University of the Basque Country UPV/EHU, 48940 Leioa, Bizkaia, Spain; raquel.hernaez@ehu.eus; 2 Cornea Division, Stein Eye Institute, University of California, Los Angeles, CA 90095, USA; s.gonzalez@jsei.ucla.edu (S.G.); deng@jsei.ucla.edu (S.X.D.); 3 Department of Applied Mathematics and Statistics and Operational Research, Biocruces Bizkaia Health Research Institute, University of the Basque Country UPV/EHU, 48940 Leioa, Bizkaia, Spain; arantza.urkaregi@ehu.eus; 4 Clinical Epidemiology Unit, Cruces University Hospital, Biocruces Bizkaia Health Research Institute, 48903 Barakaldo, Bizkaia, Spain; joseignacio.pijoanzubizarret@osakidetza.eus

**Keywords:** limbal epithelial stem/progenitor cells (LESC), limbal stem cell deficiency (LSCD), cell therapy, xenogeneic-free cell expansion, human serum (HS), serum derived from plasma rich in growth factors (s-PRGF), human amniotic membrane (HAM), explants culture, multivariate statistical analysis, principal component analysis (PCA) combined with clusters analysis, partial least squares discriminant analysis (PLS-DA)

## Abstract

Transplantation of human cultured limbal epithelial stem/progenitor cells (LESCs) has demonstrated to restore the integrity and functionality of the corneal surface in about 76% of patients with limbal stem cell deficiency. However, there are different protocols for the expansion of LESCs, and many of them use xenogeneic products, being a risk for the patients’ health. We compared the culture of limbal explants on the denuded amniotic membrane in the culture medium—supplemental hormone epithelial medium (SHEM)—supplemented with FBS or two differently produced human sera. Cell morphology, cell size, cell growth rate, and the expression level of differentiation and putative stem cell markers were examined. Several bioactive molecules were quantified in the human sera. In a novel approach, we performed a multivariate statistical analysis of data to investigate the culture factors, such as differently expressed molecules of human sera that specifically influence the cell phenotype. Our results showed that limbal cells cultured with human sera grew faster and contained similar amounts of small-sized cells, higher expression of the protein p63α, and lower of cytokeratin K12 than FBS cultures, thus, maintaining the stem/progenitor phenotype of LESCs. Furthermore, the multivariate analysis provided much data to better understand the obtaining of different cell phenotypes as a consequence of the use of different culture methodologies or different culture components.

## 1. Introduction

The corneal epithelium is a stratified squamous epithelium from which superficial terminal cells are naturally shed. The continuous renewal of the corneal epithelium is provided by a population of stem/progenitor cells located in the transitional zone between cornea and conjunctiva, known as the sclerocorneal limbus [1,2,3,4]. The specific location of limbal epithelial stem/progenitor cells (LESC) is thought to be at the limbal epithelial crypts in the palisades of Vogt [5,6].

When the limbal area is partially or totally damaged, limbal stem cell deficiency (LSCD) occurs, a condition characterized by corneal ingrowth of conjunctival epithelium, neovascularization, recurrent epithelial defects, scarring, pain, and reduced vision [7]. In such cases, grafting of limbal tissue or ex vivo expanded human LESCs can restore the structural and functional integrity of the corneal surface [6,8].

Many protocols of ex vivo expansion of LESCs have been developed in the past years [6,9,10]. These culture protocols could be divided into two main groups: the ones that use the explants culture system or, alternatively, the ones in which single-cell suspensions obtained by a different kind of enzymatic isolation are used. Additionally, these two main culture systems have some variants. Thus, explants or cell suspensions could be cultured on top of different type of supports, such as fibroblast feeder cells from different origin (murine and human), human mesenchymal stem cells as feeder cells or in 3D culture, human amniotic membrane (denuded or intact), treated plastic or different carriers, such as fibrin and collagen matrixes, or lens capsules, among others [11,12,13,14,15,16,17]. Traditionally, the majority of the possible variants of both methods still used xenogenic or undefined culture components, including the murine feeder cells or fetal bovine serum (FBS) as a supplement for the culture medium [10]. It is well known that there are numerous drawbacks and limitations in the use of animal-derived products, such as FBS, in stem cell therapy. In clinical use, the inclusion of FBS for the expansion of the stem cells could bring immunological risks [18,19,20,21,22,23].

In order to ensure the patient’s safety in all procedures, but still obtaining a large number of stem cells in the shortest possible time, the replacement of all xenogenic reagents is postulated, mainly the FBS, which is widely used as a supplement of the culture medium. Different regulatory agencies (European Medicines Agency and U.S. Food and Drug Administration) advise reducing the use of FBS and other animal products to avoid the potential risk of transmission of animal viruses, prions, and foreign proteins than can induce xenogenic immune responses [22,24]. Human serum could be the first candidate to replace the FBS as a supplement of the medium. Thus, previously several authors have introduced the use of human autologous serum or human platelet lysate as an alternative in order to avoid the use of FBS in the LESC culture system but maintaining the complex media traditionally used in the culture of these cells, obtaining similar results to those that used FBS [25,26,27,28,29]. In addition to replacing animal serum, other authors have gone a step further by including culture systems in which it has been tried to avoid the use of all xenogenic components that might be added, such as the 3T3 murine fibroblast feeder-layer (being substituted by a human amniotic membrane, a fibrin gel, biocompatible scaffolds, or by different human cell types), the cholera toxin, or the bovine pituitary extract (being substituted by xeno-free commercial or modified SHEM (supplemental hormone epithelial medium) media supplemented with or without human serum) with promising results [12,23,30,31,32,33,34].

There are marked variations in the protocols of preparing human blood derivatives. These different methodologies are able to produce significant impacts on the concentration of platelets, leucocytes, and plasma protein content, which could be turned into different responses in the expansion of stem cells in culture [22]. In this way, our group previously showed that human serum was able to support the in vitro proliferation of the human corneal epithelial (HCE) cell line [35]. Based on the encouraging results that we previously obtained in in vitro and in vivo studies using two types of human blood derivatives, s-PRGF (serum derived from plasma rich in growth factors) and HS (human serum, commonly used in an autologous manner in the sanitary environment) [36,37,38], we hypothesized that FBS could be replaced by these human sera for the expansion of LESCs.

Besides, with the aim of avoiding the use of animal products, the human amniotic membrane (HAM) has been widely used as a substrate for ex vivo-cultured LESCs before transplantation [9,39,40,41]. HAM acts both as a substrate and a carrier for the cultured cells and can retain the stem cell-like phenotype. In addition, HAM has been shown to be low immunogenic and to have anti-inflammatory, anti-fibrotic, anti-angiogenic, as well as anti-microbial [42,43].

In the present study, we compared the effect of HS or s-PRGF to that of FBS as a supplement of the culture medium on the cell growth and phenotype of LESCs ex vivo expanded on denuded HAM. We also characterized the content of each blood preparation in some growth factors and other bioactive molecules, and we studied its relation with the growth and phenotype of LESCs in the different culture conditions.

## 2. Results

### 2.1. Cell Size

**Figure 1:**
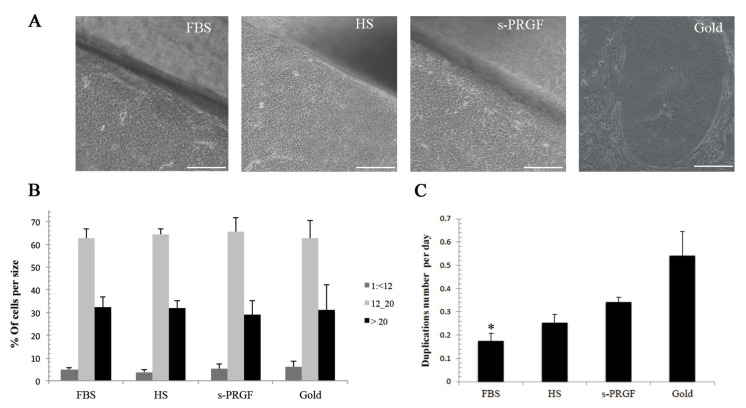
LESCs cultured following the gold standard or explants culture methodologies with FBS or human HS and s-PRGF sera: (**A**) Phenotype of cell cultures; (**B**) Percentage of cells corresponding to each cell size; (**C**) Cell growth rate represented as the number of cell duplications per day. * Indicates significant differences between the gold standard culture and the indicated treatment. (* *p* < 0.05). Scale bar in phase-contrast microscopy images, 100 micrometers. LESCs: limbal epithelial stem/progenitor cells; FBS: fetal bovine serum; HS: human serum; s-PRGF: serum derived from plasma rich in growth factors.

No significant differences were found between groups in the distribution of cells according to their size. Cells from the explants cultured with human s-PRGF and HS presented a similar distribution in cell size in comparison with the ones cultured with FBS (Figure 1A,B). Concerning cultures treated with FBS but using two different methodologies of culture—the gold standard treatment (isolated cells cultured with FBS onto murine inactivated fibroblast) and explants cultured with FBS—both showed a similar number of cells with size 2 and 3, but a higher number of size 1 cells (cells smaller than 12 µm) for the gold treatment (6.2%) than for the explants methodology (4.9%). When analyzing the different pools of human sera, HS1 and sPRGF2 showed a similar number of the smallest sized cells to the gold standard treatment (6.4% and 6.3%, respectively), whereas the other treatments showed fewer cells (1.6% for explants cultured with HS2 and 4.4% for explants with s-PRGF1) (Supplementary Table S1).

### 2.2. Cell Growth Rate

The cultures under the explants methodology treated with human sera grew faster than the cultures under the explants methodology treated with FBS. Thus, cultures under s-PRGF treatment took a media of only 2.9 days in duplicating their number of cells, and 4.38 days the ones cultured with HS, while the ones treated with FBS took 6.15 days (Figure 1C). There were no significant differences between none of the treatments, but a *p*-value = 0.063 was observed when comparing s-PRGF versus FBS treatments. Cells cultured with the gold standard treatment showed the shortest population doubling times (2.03 days), differences being statistically significant with respect to explants cultures treated with FBS (*p* = 0.032). Attending to separate pools, the only treatment that showed a similarly fast cell growth to the gold standard treatment was the s-PRGF1 treatment (2.49 days) (Supplementary Table S1).

### 2.3. LESC Protein Marker Analysis

**Figure 2:**
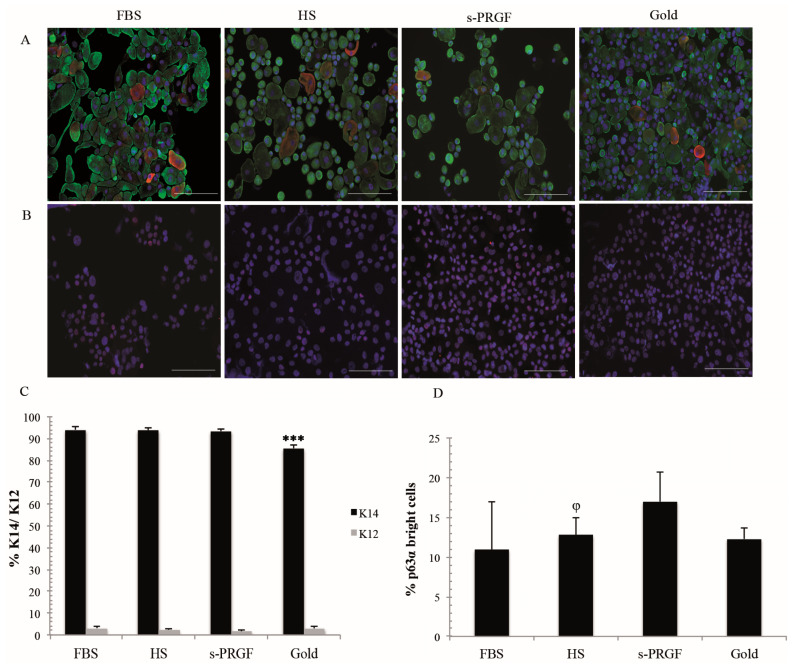
Protein expression profiles of several cell markers in LESCs obtained from explants and gold standard cultures treated with FBS or two different human sera: (**A**) Double immunostaining for K14 (green) and K12 (red) cytokeratins; (**B**) Immunostaining for p63α; (**C**) Percentage of K14 and K12 positive cells; (**D**) Percentage of p63α bright cells. *** Indicates significant differences between the gold standard culture and all the other treatments (*p* < 0.001), and ^φ^ between FBS and the indicated treatment (*p* < 0.05). Scale bar for fluorescence images, 100 micrometers.

We studied the limbal stemness-associated markers—K14 and ΔNp63α—along with the corneal epithelial differentiation marker K12. Cultures maintained with human sera, and especially with s-PRGF, were the ones with the lowest number of K12 positive cells (average of 1.7% to an average of 2.4%) (Figure 2A,C). Moreover, we found a similar number of K12 positive cells in both FBS treatments, that is, in the isolated-cells (3.0%) and explants culture (3.1%) methodologies. No significant differences between LESCs cultured under any methodology and with HS, s-PRGF, or FBS on the percentage of K12 positive cells were found (*p* > 0.05).

For the stemness-associated marker K14, the gold standard treatment showed lower positive cells (85.6%), being differences significant (*p* < 0.001) with respect to the FBS (94.1%) as well as to the s-PRGF (average of 93.4%) and HS (average of 94.0%) treatments. In addition, all cultures under the explants methodology showed a similar percentage of K14 positive cells, with no significant differences between them.

For the stemness-related marker ΔNp63α, only the p63α positive bright cells were quantified. For the explants methodology, the percentage of p63α bright cells was higher in LESCs cultured with s-PRGF (average of 17.0%) or HS (average of 12.8%) than in cells cultured with FBS (10.9%) (Figure 2B,D). We found significant differences between cultures treated with FBS and HS (*p* = 0.017) but not with s-PRGF (*p* = 0.064) or the gold standard culture methodology (12.3%) (n.s.). With respect to different pools, cultures treated with s-PRGF2 and HS2 showed the highest number of p63α positive cells (20.9% and 15.3%, respectively) (Supplementary Table S1).

### 2.4. Gene Expression Analysis

**Figure 3:**
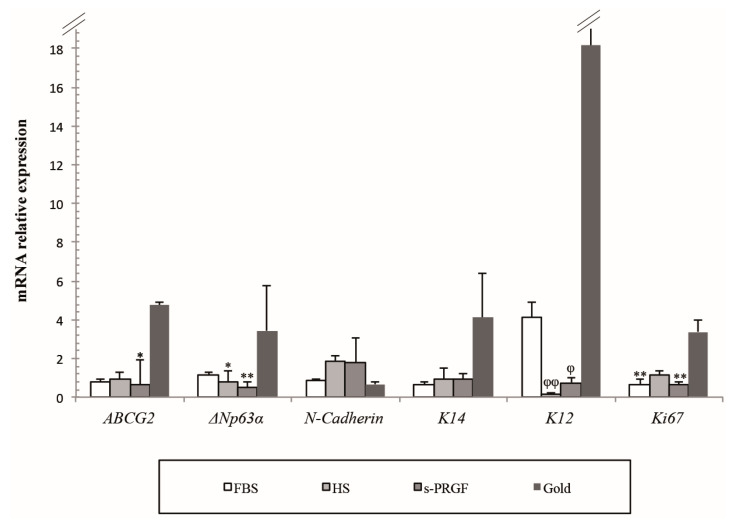
Relative mRNA expression of several cell markers in LESCs obtained from explants and gold standard cultures treated with FBS or two different human sera. * Indicates significant differences between the gold standard culture and the indicated treatments, and ^φ^ between the FBS and the indicated treatments. (* *p* < 0.05; ** *p* < 0.01) (^φ^ *p* < 0.05; ^φφ^ *p* < 0.01).

We analyzed by qPCR the expression level of several cell markers attributed to limbal stem/progenitor cells, such as *ABCG2*, *ΔNp63α*, *N-Cadherin*, and *K14*, as well as the *K12* marker of differentiated corneal epithelial cells. To evaluate cell proliferation, we studied the expression of the *Ki67* marker (Figure 3).

We observed a notable difference in the expression of the majority of the cell markers studied in accordance with the culture methodology used. Thus, LESCs cultured under the gold standard treatment, that is, isolated-cells cultured with FBS, showed the highest expression for the majority of cell markers except for *N-Cadherin*; the fold-change (FC) expression being of a minimum of 5 for *ABCG2*, 3 for *ΔNp63α*, and 4 for *K14* putative stem cell markers when compared with all other cultures (explants cultures). Significant differences were found between a gold standard and explants cultures treated with s-PRGF for *ABCG2* (*p* = 0.047) and between the gold standard and explants cultures treated with both human sera for *ΔNp63α* (*p* = 0.022 for HS and *p* = 0.002 for s-PRGF) putative stem cell markers. The gold standard treatment showed the lowest expression for the *N-Cadherin* marker. When analyzing the expression of LESCs cultured using the explants methodology, the expression of *K14* and, especially *N-Cadherin* cell markers, was higher in cultures treated with human sera than with FBS. However, there were no statistically significant differences for any of the putative stem cell markers between cultures under the explants methodology.

For the differentiation cell marker *K12*, the FC expression of cultures treated with the gold standard was of a minimum of four with respect to explants cultures treated with FBS and of 22 when compared with explants cultures treated with human sera. As expression for the *K12* marker was not very reproducible between replicas treated with the gold standard treatment, significant differences were only found between FBS and both human sera (*p* = 0.001 for HS and *p* = 0.021 for s-PRGF).

The proliferation marker *Ki67* showed a minimum FC of three for the gold standard treatment with respect to the explant cultures. Differences were very significant between the gold standard treatment and explants cultures treated with FBS and s-PRGF (*p* = 0.003 and *p* = 0.002, respectively). There were no significant differences between cultures under the explants’ methodology.

### 2.5. Quantification of Growth Factor and Cytokines in Human Sera

To characterize the sera and further investigate if some of the components in them could be involved in the differentiation status of limbal cultures, we measured the concentrations of several growth factors and cytokines in two different pools of each human serum.

We detected higher concentrations of epidermal growth factor (EGF), hepatocyte growth factor (HGF), fibroblastic basic growth factor (FGFb), and keratinocyte growth factor (KGF) in HS than in s-PRGF (Table 1) with significant differences between both sera only for FGFb (*p* = 0.040). Moreover, pool number 2 of both sera showed higher concentrations than pool number 1.

The concentrations of platelet-derived growth factor AB (PDGF-AB), transforming growth factor-beta 1 (TGF-β), vascular endothelial growth factor (VEGF), and fibronectin were very similar between both sera. Significant differences were only found for the fibronectin between HS2 and s-PRGF2 (*p* = 0.003).

Very low concentrations of the proinflammatory cytokines—tumor necrosis factor-alpha (TNF-α) and interferon-gamma (IFN-γ) were measured, which were very similar between both sera.

With respect to the presence of neurotrophic factors and neuropeptides, the concentrations of nerve growth factor (NGF) and insulin-like growth factor 1 (IGF-1) were similar between both human sera. However, the concentrations of glial cell line-derived neurotrophic factor (GDNF) and substance P (SP) were higher in HS than in s-PRGF, showing statistically significant differences between both sera (*p* = 0.032 for GDNF and *p* = 0.012 for SP). Attending to different pools, HS1 contained higher concentrations of SP than s-PRGF1, showing significant differences (*p* = 0.001).

### 2.6. Correlations

Some correlations were found between the different variables analyzed (Table 2 and Table 3). They have been discussed in the Discussion chapter.

### 2.7. Multivariate Analysis of Data

With the aim of comparing the behavior of cultures treated with the different treatments, we first performed a principal component analysis (PCA), taking as variables the stemness, differentiation, and proliferation markers, both at the level of protein and mRNA. Four main components were obtained that explained 75% of the total variance (Supplementary Figure S1).

**Figure 4:**
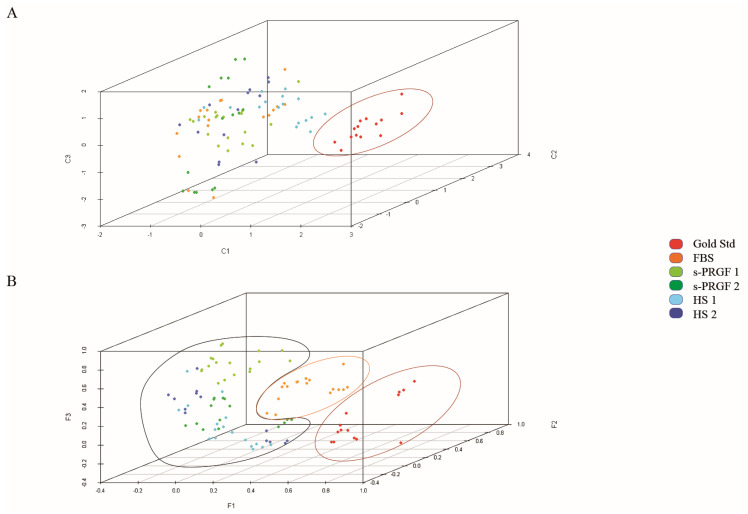
Representation of all samples in three components obtained by (**A**) Principal component analysis (PCA); (**B**) Partial least squares discriminant analysis (PLS-DA). Colors indicate treatments. Cultures following the gold standard culture methodology are separated from cultures following explants culture methodology.

The different behavior of the gold standard treatment was observed in comparison to the rest of the treatments, which followed the explants culture methodology (Figure 4A), meaning that the expression of cell markers was very different in LESCs following the gold standard culture or explants culture methodology. The separate classification of the gold standard treatment was ratified by analyzing the samples using the partial least squares discriminant analysis (PLS-DA) (Supplementary Table S2). Moreover, we could clearly distinguish that cultures treated with human sera behaved more similarly between them and didn’t mix with cultures treated with FBS, although using the same explants culture methodology (Figure 4B). The PLS-DA analysis presented differences in the prediction capacity of each of the treatments (Supplementary Figure S2), while the global prediction capacity provided by the multi-class area under the curve (AUC) for the receiver operating characteristic curve or ROC curve was 0.885.

In our study, there were several human factors related to cell culture. Thus, we used three different corneas that, in the explants culture methodology, were cultivated on a different denuded human amniotic membrane (HAM) in each case. Each group of cornea-HAM was treated with the four human sera or with FBS. We could not observe a clear classification due to the cornea-HAM factor, suggesting that the type of treatment was more important for the classification of the samples than the corneas or HAMs themselves (Supplementary Figure S3).

In order to detect the differences between cultures following the explants culture methodology, we removed the samples corresponding to the gold standard treatment from the analysis and redid the PCA analysis. We also obtained main four factors that explained 72.4% of the total variance (Supplementary Figure S4). The three-dimensional graphic representation (Supplementary Figure S5) showed that cultures treated with FBS were grouped and differed from treatments with human sera, which appeared mixed. We obtained the same results when we applied the PLS-DA analysis.

**Figure 5:**
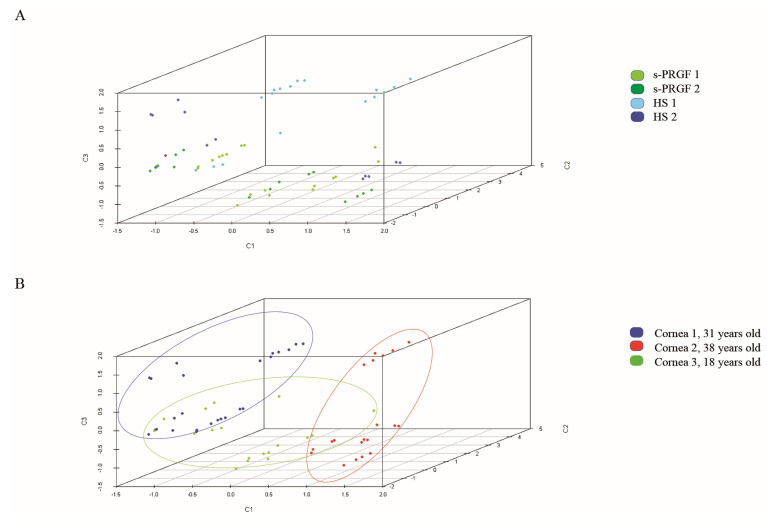
Representation of samples treated with human sera in three components obtained by PCA analysis. Colors indicate (**A**) treatments; (**B**) group of cornea-HAM (human amniotic membrane). Samples were classified mainly by cornea-HAM and not by treatment.

Then, to appreciate differences between LESCs cultured with the different human sera, we removed the samples corresponding to the explants culture methodology treated with FBS and did the PCA analysis again. The main four factors obtained, which explained 75% of the total variance (Supplementary Figure S6), showing that this time the type of sera and culture methodology were more homogeneous between them, and the samples showed higher classification as a function of the cornea used for cell cultures (Figure 5A,B). Moreover, the more aged cornea (38 years old. donor, cornea n.2) was classified apart from the other two (31 years old donor, cornea n.1; 18 years old donor, cornea n.3). When analyzing the variables corresponding to the different components of the PCA analysis, the younger corneas (n. 1 and 3) showed a higher expression of the protein p63α than the older cornea n.2.

**Figure 6:**
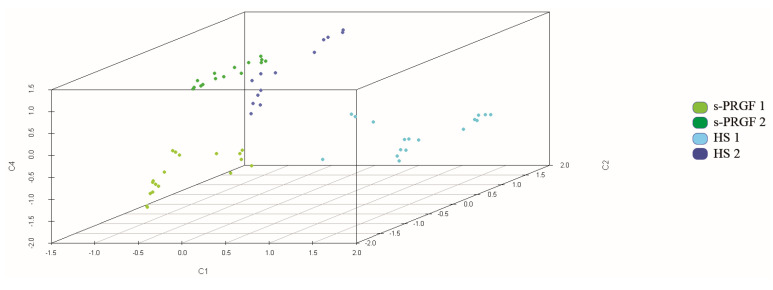
Representation of samples treated with human sera in three components obtained by PCA analysis. The analysis also included as variables some molecules measured in sera (glial cell-derived neurotrophic factor (GDNF), substance P (SP), epidermal growth factor (EGF)). Colors indicate treatments. Samples were well classified by treatment.

Finally, we repeated the PCA analysis only for the samples treated with human sera, but we also included as variables in the analysis three of all the growth factors measured in the several sera—the neurotrophic GDNF and SP factors, whose content showed statistically significant differences between the different human sera, and EGF, which is crucial for the survival of epithelial cells. We obtained the main five factors that explained 82% of the total variance (Supplementary Figure S7). The three-dimensional graphic representation (Figure 6) showed that cultures treated with the different human sera classified separately and showed differences between them.

Next, we conducted a hierarchical cluster using as variables the five-factor scores obtained in the last PCA analysis, and we obtained 12 clusters (Supplementary Figure S8). The 12-clusters dendrogram showed that there was a classification based on the pool of serum, which means that, in our study, serum donors (that is, the concentrations of the different bioactive molecules with which each individual contributes to the serum) were more important to define cell phenotypes than the own methodology of elaboration of the serum (HS or s-PRGF) or the characteristics of the cornea and HAM used in the cultures.

On the other hand, in the dendrogram of 12 classes, each class corresponded practically to a type of serum combined with a specific cornea-HAM set (Table 4). In this way, the analysis of the characteristics of each class allowed us to see the differences in treating cells from the same cornea with different human sera or to see if the same serum had different effects when added to cells obtained from different corneas. Thus, for cells from a specific cornea cultured with the different human sera, the expression of the variables analyzed was very different depending on the serum used (Table 4). Similar variability was obtained when a specific treatment was added to cells from different corneas.

Taking into account all PCA analysis done, a positive correlation between PCR-*K14* and PCR-*ABCG2*, as well as a negative correlation between ICC-K14 and ICC-K12 variables, was always maintained. So, analyzing only one of the variables in the tandem, results would apply for the other one.

When the gold standard treatment was excluded from the PCA analysis (Supplementary Figure S4), cultures following the explants culture methodology showed that the expression of PCR-*K14* and PCR-*ABCG2* was correlated with a negative expression of p63α at the protein level (ICC-ΔNp63α). In addition, a negative correlation between PCR-*Ki67* and PCR-*K12* was observed. So, cultures with the high expression for PCR-*K12* would show low expression of PCR-*Ki67*.

## 3. Discussion

As the transplantation of limbal epithelial stem cells (LESC) has been proved as an effective treatment of limbal stem cell deficiencies [44,45], different approaches to culture LESCs for transplantation have emerged in the last two decades. Trying to avoid xenogenic components during in vitro expansion of LESCs, other authors have previously demonstrated that the culture of LESCs could be supported by a mixture of complex medium supplemented with human serum or human platelet lysate [23,27,29,31,34,46,47,48,49], or even in a simple medium containing human serum and no other supplements [9]. The present study indicated that LESCs could be expanded ex vivo on denuded HAM using two types of human serum, s-PRGF and HS, instead of FBS in the SHEM complex medium. This culture system using denuded HAM as a substrate, which supports the growth of well-stratified and differentiated limbal cells [16,40,50,51], together with the substitution of FBS for human serum reduces the use of animal-derived products. In addition, the explants culture methodology could provide the original niche with all the surrounding supporting cells, including limbal and stromal cells, which are thought to help in the maintenance of stem/progenitor cells in culture [52,53]. Additionally, this study tried to elucidate what are the factors in the serum that could be related to the maintenance of the differentiation status and the proliferation of LESCs in vitro.

It is well known that the different types of methodologies used for cell culture influence the behavior and expression of markers in cultured cells. In the present work, we demonstrated this fact using multivariate statistical analysis. Our results showed that the expression of cell markers in LESCs cultured as enzymatically isolated cells onto a feeder layer of mitotically inactivated 3T3 was different from that of LESCs cultured following the explants culture methodology on HAM. Even more, using the explants culture methodology, cultures treated with FBS classified apart from cultures treated with human sera. This mathematical approach supported that signaling molecules released from 3T3 or from stromal cells from explants, as well as contents in growth factors in the different sera, could be responsible for the changes in the expression pattern of cell markers of LESCs.

Moreover, when LESCs were cultured as explants and treated only with human sera, so culture conditions were more similar between them, we detected differences in the expression of cell markers due to the use of several different corneas. The 38 years old cornea classified separately from corneas aged 31 and 18 years, the latter ones expressing higher protein p63α levels. The effect of age on the structural and phenotypic characteristics of the human limbal niche has been demonstrated [8], the colony-forming efficiency of the limbal cultures being significantly reduced from donors of 30 years and over. These data suggested that LESCs harvested from younger donors might be more suitable for cultured LESC therapy production.

Multivariate analysis also showed several correlations between the variables studied in our experimental system. In this sense, we observed, in all the PCA analyses done, a strong correlation between the mRNA level of *K14* and *ABCG2* as well as between the protein level of K12 and K14. So, analyzing one of the variables would be sufficient to have the information of both correlated markers. Because of that, using only some of the cell markers studied could be enough to describe cell phenotypes, which, in turn, would reduce the redundancy of experimental analysis.

Besides other cellular markers of LESCs, the cellular size is an important feature that can be used to identify or isolate these cells. According to Romano [54], there is a marked difference among the limbal basal epithelial cells (where limbal epithelial progenitor cells exist) and the central corneal basal epithelial cells. Thanks to in vivo confocal microscopy, they found that the size of the basal cells of the limbus was 10.1 ± 0.8 µm, whereas the basal cells of the central epithelial cornea had a size of 17.1 ± 0.8 µm. Our data revealed that 5.4% of the cells cultured in explants system with s-PRGF presented a size smaller than 12 micrometers, followed by HS (3.8%) and FBS (4.9%), which could represent the limbal stem cell population. Our data suggested that both human sera could retain small cells like progenitor limbal cells, being the s-PRGF the serum with more percentage of this kind of cells in the explants culture system.

Although there are not definitive protein markers for identifying LESCs, several proteins have been suggested as potential markers. The percentage of p63α positive bright cells has been suggested to be indicative of the clinical outcome after transplantation to patients with LSCD [45]. In our culture system, the average of cells expressing the protein p63α was higher in explants cultured with both human sera than with FBS or in the gold standard culture system. The expression of the keratin 14 marker was also higher in all explants cultures than in the gold standard treatment. These results showed the stemness potential of cultures, using the explants culture methodology, which were treated with human sera. On the contrary, at the mRNA level, the expression of the putative stem cell markers—*ΔNp63α* and *ABCG2*—was significantly lower for the explants cultured with the human sera than for the gold standard treatment. In fact, the PCA analysis of explants treated with human sera showed a negative correlation between mRNA levels of *K14* and *ABCG2* with respect to the protein level of p63α. It was also corroborated by the Spearman test (correlation coefficient −0.74, *p* = 0.006 between mRNA *K14* and protein p63α).

When we attended the markers of corneal epithelial cells, such as K12, we observed a higher expression in the culture systems supplemented with FBS in comparison to the ones with human sera. It has been observed by other authors, too [9,25]. The same occurred at the mRNA level, being the expression of *K12* significantly higher for treatments using FBS. PCA analysis of explants cultures showed an inverse correlation for mRNA levels of *Ki67* and *K12*, indicating that cultures with high expression of mRNA *K12*, like the ones treated with FBS, would show lower expression of mRNA *Ki67*. In addition, for explants cultures treated with human sera, the Spearman test showed inverse correlations between the *K12* mRNA expression and the cell growth expressed as days required for cell duplication (correlation coefficient −0.8, *p* = 0.01). Thus, for cultures with fast cell growth, such as the PRGF treatment, with fewer days needed for cell duplication than the HS treatment, the expression of *K12* mRNA was higher. We hypothesized that when cells with such a high growth rate reach the confluence in a short period of time, they start to differentiate, maybe due to stratification, and reduce proliferation rate, showing at the mRNA level, at the time of being collected, higher production of differentiation markers and lower production of the proliferation marker *Ki67*. Furthermore, also for the explants cultures methodology, PCA and Spearman analysis showed correlations between the mRNA level of *Ki67* and the intermediate cell size (12–20 µm), pointing to these cells as the most proliferative ones. Maybe, they could be the transient amplifying cells’ population.

Trying to elucidate which were the factors present in the human sera that could be involved in the maintenance of the multipotency of the LESCs, our group performed ELISA assays to assess in each human serum the concentration of relevant growth factors and biologically active molecules. Growth factors are a group of substances, mainly proteins, secreted by cells, which stimulate a specific cell receptor and affect cell functions, such as migration, proliferation, and differentiation, during development and tissue growth [55]. The same growth factor can have antagonistic functions depending on the extracellular microenvironment. In this way, they could stimulate stem/progenitor cells either by increasing their survival or their proliferation rate [56].

In cultures treated with human sera, we observed moderately strong correlations for the content of human sera in PDGF-AB and GDNF growth factors with respect to the duplication time of cultures (correlation factor −0.75 and 0.75, respectively, *p* < 0.05) and the mRNA expression of *K12* (correlation factor 0.73 and −0.73, respectively, *p* = 0.007). Inverse associations of *K12* mRNA with GDNF (and SP) were also described in the PCA analysis. Moreover, a strong inverse correlation (−1) was observed between the concentration of PDGF-AB and GDNF (*p* < 0.01). Thus, the previously discussed faster cell growth (lower duplication times) and higher mRNA expression levels of *K12* could be stimulated by high concentrations of PDGF-AB and low concentrations of GDNF. PDGF is a disulfide-linked dimer composed of five different peptide chains, with the AA, AB, and BB being the most expressed isoforms in the ocular surface. They regulate the migration and proliferation of keratocytes [57,58]. PDGF also promotes in vitro chemotaxis of epithelial cells in the presence of fibronectin [59] and stimulates epithelial wound healing in vivo [60]. Fibronectin is an important glycoprotein present in the extracellular matrix (ECM). The ECM of the limbal niche is a dynamic microenvironment that provides mechanical and structural support for stem cells but also regulates cellular functions. It has been shown that fibronectin enhances proliferation and regulates stemness of rabbit limbal epithelial stem cells [61]. In addition, during wound healing, fibronectin/fibrinogen receptors are up-regulated on epithelial cells, which migrate over the bare wound [62]. Therefore, the high concentrations of fibronectin and PDGF found in blood derivatives could favor cell migration, inducing the epithelial cells to move from the explants to different areas on the top of the HAM, while contributing to promoting LESCs’ proliferation and preservation of their stemness properties.

GDNF, a distant member of TGF-β superfamily of growth factors, is a neurotrophic factor. Although neurotrophic factors are defined as polypeptides that maintain neuronal cells, they have been reported to possess a range of functions in promoting survival and self-renewal of stem cells outside the nervous system, like spermatogonial stem cells [63]. Any dysfunction on corneal nerves could cause the breakdown of corneal epithelium and ulceration [64] and significantly reduce the quantity and normal function of corneal stem/progenitor cells [65]. GDNF and its specific receptor GFRα-1 are expressed in the basal area of the limbal epithelium [64,66]. Substance P (SP) is an 11_amino acid neuropeptide from the tachykinin family that acts as a neurotransmitter-mediating nociceptive transmission. In the cornea, SP has been detected in the nerve fibers of naïve cornea [67,68], and it is able to activate the epidermal growth factor receptor (EGFR) signaling pathways, among others [69,70]. SP has a role in corneal homeostasis. It promotes corneal epithelial cell proliferation [71,72], migration [73], the expression of intercellular junctional molecules, contributing to the maintenance of the barrier function of epithelial cells [74], and inhibits apoptosis [70]. The combination of IGF-1 and SP also increases wound closure in patients with neurotrophic keratopathy [75,76]. According to our results, HS showed a significantly higher concentration of GDNF and SP than s-PRGF (*p* < 0.05). Spearman and PCA analysis revealed a strong positive correlation between the concentration of GDNF and SP in our human sera (correlation coefficient: 0.800, *p* = 0.002), as well as a positive correlation between the concentration of both and the mRNA level of the proliferation marker *Ki67*, and an inverse correlation between the concentration of both and the mRNA level of *K12* (correlation coefficient: −0.734 and −0,648 respectively, *p* < 0.05), suggesting a role for both factors in the epithelial cells’ proliferation and the retention of the undifferentiated status of the culture. In addition, the Spearman correlation test revealed a total inverse correlation between the concentration of the neuropeptide SP and TNF-α (correlation coefficient: −1.0; *p* < 0,001). The inhibition of TNF-α secretion by SP in a retinal damage animal model has been described [77], indicating a possible anti-inflammatory effect for SP.

Another neurotrophic factor, NGF, stimulates the proliferation, migration, and differentiation of corneal epithelial cells in culture and has been shown to promote healing of corneal ulcers and impaired epithelial defects in several case studies and clinical trials [78,79]. NGF, as well as its high-affinity receptor tropomyosin kinase A (TrkA), is mainly expressed in the limbal basal epithelial. Recently, it has been shown that NGF is a promoter of proliferation and maintenance of the limbal stem cell phenotype [80]. According to our results, HS and s-PRGF presented very similar concentrations of NGF, indicating that both have the capability to retain the pool of limbal stem cells present in the corneal explants. All these neurotrophic factors and neuropeptides present in human sera could be modulating the behavior of limbal cultures in terms of proliferation, migration, stemness, and differentiation of epithelial cells as well as avoiding inflammatory processes.

With respect to the characteristics of the human sera depending on the methodology of production, we detected higher concentrations of EGF, HGF, FGFb, KGF, GDNF, and SP in HS than in s-PRGF, indicating that the methodology of production of the serum influences the content of these factors. When we studied correlations between these growth factors, we found a 100% positive linear association according to Spearman test between EGF, HGF, and KGF (correlation coefficient: 1.0 or; *p* < 0.001), meaning that the three growth factors varied in the same manner in each serum. It has been described that diminished HGF and EGF secretion by diseased lacrimal tissue in keratoconjunctivitis sicca could have a role in the pathophysiology of the disease and that the levels of EGF, HGF, and KGF mRNAs increase in lacrimal gland tissue in response to corneal epithelial wounding [57,81], pointing to a common function for them. Other strong associations between the content in growth factors in the human sera have already been discussed.

With respect to the production protocol to obtain the human sera, PLS-DA analysis showed that samples treated with s-PRGF classified better than those treated with HS, suggesting that cells cultured with s-PRGF behave in a more reproducible manner. Reproducibility is an important factor for cell therapy. On the other hand, HS showed to have higher concentrations of the neurotrophic factor GDNF and the neuropeptide SP than s-PRGF. Altogether, these data show again that depending on the way of producing the serum, the content in growth factors and other bioactive molecules differ and influence the evolution of cell cultures. So, it would be very interesting to know which of the several characteristics observed in the different types of serum would be more decisive for the outcomes of the LESC transplantation.

In addition, the pool number 2 of both serum types showed higher concentrations than the pool number 1 for EGF, HGF, KGF, TGF-β, and IFN-γ, whereas for SP, the pool number 1 showed higher concentrations than the pool number 2, suggesting that the content of all these growth factors depends on their content in donors’ blood, reflecting the variability between individuals.

## 4. Materials and Methods

### 4.1. Human Sclerocorneal Tissue

Human sclerocorneal tissue was obtained from Illinois Eye Bank (Watson Gailey, Bloomington, IL, USA) and Lions Eye Institute for Transplant and Research (Tampa, FL, USA) from 20 to 65 years old healthy donors. Experimentation on human tissue was performed in accordance with the tenets of the Declaration of Helsinki. The experimental protocol was approved by the University of California Los Angeles Institutional Review Board. The tissues were preserved in Optisol solution (Chiron Ophthalmics, Inc., Irvine, CA, USA) at 4 °C for less than 72 h. For the cell culture, the death-to-preservation time was less than 8 h.

### 4.2. Preparation of Blood-Derived Products

Blood from 9 healthy volunteers was collected by venipuncture (age range 25–60 years) in accordance with the tenets of the Declaration of Helsinki and with proper informed consent. The procedures were approved by the Ethics Committee of the University of the Basque Country UPV/EHU (CEISH/342/2015/ANDOLLO VICTORIANO, 16 April 2015). All volunteers were healthy and not taking any medication. The blood sample from each volunteer was divided and processed by 2 previously described methods [35] to obtain the corresponding blood derivatives. Briefly:

1. Human serum (HS): Spontaneous coagulation for 2 h at room temperature followed by centrifugation at 1000 g for 15 min. The complete supernatant fraction was collected.
2. Serum derived from plasma rich in growth factors (s-PRGF): Centrifugation at 460 g for 8 min, followed by a collection of the complete supernatant fraction and induction of clot formation by adding calcium chloride (Braun, Barcelona, Spain) at a final concentration of 22.8 mM in the absence of red and white blood cells. After 2 h at 36 °C, the clot was retracted, and the supernatant was collected.

For blood collection, we used tubes with sodium citrate as an anticoagulant for s-PRGF processing or without anticoagulant for HS processing. The complement factors of all blood derivatives were inactivated at 56 °C for 30 min. Afterward, we pooled samples from the different volunteers to obtain representative blood preparations that provided reproducible results and minimized inter-individual variability. These pools were stored at −20 °C until its use for the in vitro assays.

### 4.3. Amniotic Membrane Preparation

In accordance with the tenets of the Declaration of Helsinki and with proper informed consent, human amniotic membranes were obtained at the time of Cesarean section. Under sterile conditions, all the membranes were washed with sterile phosphate-buffered saline (PBS). Then, they were cleaned from blood clots, cut into pieces, and stored at −80 °C in an appropriate cryopreservation medium (DMEM, ATCC, Manassas, VA, USA). Immediately before use, the pieces of the amniotic membrane were thawed and washed once in Dulbecco’s Phosphate-Buffered Saline (DPBS, Lonza, Basel, Switzerland) and then twice in a small volume of freshly opened Versene (0.02% EDTA in PBS) (Gibco; Thermo Fisher Scientific, Inc., Waltham, MA, USA). To loosen cellular adhesion junctions, HAM was placed in a flat large Petri dish and submerged in Versene at 37 °C for at least 2 h, until cells come off easily on their own or by gentle scraping. The pieces of decellularized HAM were then washed twice with sterile PBS, mounted on filter paper rings, creating a circle of about 30 mm of the diameter of free HAM, and placed in a well of a 6-well plate with 2 mL of DMEM overnight.

### 4.4. Tissue Explants and Limbal Epithelial Cell Isolation

In order to prepare cell cultures, at least 3 corneoscleral rims of three different donors were separated from iris, endothelium, Tenon’s capsules, and conjunctiva. Then, each rim was cut into 16 pieces of 2 × 2 mm each. Explant tissue pieces were placed with the epithelium side facing up onto denuded HAM circular pieces in 6-well plates. Only one explant piece was cultured per well.

For the isolation of epithelial cells, an explant tissue piece of 2 × 2 mm of limbal tissue from each corneoscleral rim was incubated with 2.4 U/mL of dispase II (Roche, Indianapolis, IN, USA) at 37 °C for 2 h in DMEM/F-12 medium (Life Technologies, Thermo Fisher Scientific, Inc., Waltham, MA, USA) supplemented with 5% FBS (Life Technologies). Epithelial cell sheets were then isolated by gentle scraping under the stereoscopic microscope. Single cells were obtained by incubation with 0.25% trypsin and 1 mM EDTA (Life Technologies) for 5 min.

### 4.5. Explants and Single Cell Cultures

A total of 45 explants from 3 donors (aged 31, 38, and 18 years old) were cultured on denuded HAM from another 3 donors in supplemental hormone epithelial medium (SHEM) containing DMEM/F12 supplemented with N-2 supplement (Life Technologies), 2 ng/mL epidermal growth factor (EGF, Life Technologies), 8.4 ng/mL cholera toxin (Sigma-Aldrich, St. Louis, MO, USA), 0.5 mg/mL hydrocortisone (Sigma-Aldrich), 0.5% dimethyl sulfoxide (Sigma-Aldrich), penicillin/streptomycin (Life Technologies), gentamicin/amphotericin B (Life Technologies), and 5% FBS (Life Technologies) (9 explants) or 10% HS pool 1 and pool 2 (9 and 9 explants) or 10% s-PRGF pool 1 and pool 2 (9 and 9 explants).

Single limbal epithelial cells were seeded on growth-arrested 3T3-J2 mouse fibroblasts (Howard Green Lab, Harvard Medical School, Boston, MA, USA) at a density of 300 cells/cm^2^ in 6-well plates and cultured in SHEM medium supplemented with 5% FBS. Single LESCs cultured on 3T3 cells served as the control. To prepare growth-arrested feeder layers, subconfluent 3T3-J2 cells were incubated with 4 µg/mL mitomycin C (Sigma-Aldrich) for 2 h at 37 °C. Then, they were trypsinized and seeded into a 6-well plate at a density of 3 × 10^4^ cells/cm^2^.

In all culture conditions, cells were grown at 37 °C in an atmosphere of 5% CO_2_, and the culture medium was changed every 2–3 days.

### 4.6. Analysis of Cell Growth and Cell Size

For the cell growth analysis, the total number of cells were harvested and counted from each single explant tissue piece, once the cells had reached the exterior edge of the HAM circular piece (confluence) and had been detached from the HAM by dispase/trypsin-EDTA treatment. In the case of LESC colonies grown on 3T3, cells were harvested after washing away the 3T3 monolayer mechanically by pipetting up and down the medium, and they were dissociated using trypsin-EDTA. Then, the total number of cells was counted.

The cell growth rate was measured in terms of the cell population doubling time (DT). DT was calculated as following: DT = T ln2/ln(Xe/Xb), where T is the incubation time (in days), Xb the cell number at the beginning of the incubation time, and Xe the cell number at the end of the incubation time (confluence). For the explant culture, the initial number of cells was considered to be the number of epithelial cells isolated from a 2 × 2 mm limbal tissue piece.

Cell size was measured using ImageJ software (developed by Wayne Rasband at the Research Services Branch, National Institute of Mental Health, Bethesda, MD, USA). The percentage of cells with a diameter <12 µm, 12–20 µm, or >20 µm was calculated in at least 200 cells from each culture treatment in three different experiments.

### 4.7. Real-Time RT-PCR

After culture, epithelial cells were collected, and the total RNA was extracted according to the manufacturer’s protocol (RNeasy Mini Kit; Qiagen, Valencia, CA, USA). The quantity and quality of the RNA were assessed with a NanoDrop 1000 spectrophotometer (NanoDrop, Wilmington, DE, USA). Then, the total RNAs were treated with DNase (DNA-free kit, Ambion, Austin, TX, USA) and reverse-transcribed into cDNA (SuperScript II Reverse Transcriptase, Life Technologies) according to the manufacturer’s instructions. The quantification of cDNA amplicons was assessed by fluorescence intensity by using the Kapa Sybr Fast qPCR kit (Kapa Biosystems, Woburn, MA, USA). Cycle conditions were as follows: an initial denaturing step of 5 min at 94 °C and subsequent 40 cycles of amplification in which each cycle consisted of 15 s at 94 °C, 30 s at 55 °C, and 30 s at 72 °C. To generate a dissociation curve after the amplification cycles, each sample was incubated at 95 °C for 1 min, followed by a melting curve program (55–99 °C, with a 5-s hold at each temperature). RT-qPCR was conducted independently three times with samples from each donor. The expression data (ΔCq) were generated from the average value of triplicates from each gene. The expression data obtained from the studied genes were normalized to that of the reference gene glyceraldehyde-3-phosphate dehydrogenase (GAPDH), by using the algorithms outlined by Vandesompele et al. [82]. Finally, the relative expression (2^−ΔΔCq^) for each gene was calculated by subtracting from the normalized expression data of this sample the average of the normalized expression data of all the samples, following the modification of Pfaffl [83]. The primers used for RT-qPCR are listed in Supplementary Table S3.

### 4.8. Immunocytochemistry

Cultured epithelial cells were centrifuged into cytospin slides by using a Cytofuge cytocentrifuge (Fisher Scientific, Hampton, NH) and stored at −20 °C until use. Cytospin slides were fixed with 4% paraformaldehyde at room temperature for 10 min and washed three times with PBS. They were blocked and permeabilized with PBS containing 0.5% Triton X-100 (Sigma-Aldrich) and 1% bovine serum albumin (BSA) (Sigma-Aldrich) for 30 min at room temperature. The slides were incubated with one or more primary antibodies (Supplementary Table S4) diluted in PBS with 0.1% Triton X-100 and 1% BSA overnight at 4 °C in a moisture chamber. The next day, slides were washed three times with PBS and then incubated with one or more secondary antibodies diluted in PBS with 0.1% Triton X-100 and 1% BSA at room temperature for 1 h in the dark. After, they were washed three times with PBS. Nuclei were labeled with 4 mg/mL Hoechst 33342 (Life Technologies) at room temperature for 15 min. Finally, the slides were washed five times with PBS and mounted using Fluoromount medium (Sigma-Aldrich).

Images were acquired using a Zeiss Imager.A2 fluorescent microscope (Carl Zeiss, Inc., Oberkochen, Germany) equipped with an Insight 11.2 color mosaic digital camera. The quantification of the cells that expressed a high level of p63α (p63αbright cells) was performed with the Definiens Tissue Studio software (Larchmont, NY, USA), following the previously reported criteria [84]. The number of K14 and K12 positive cells was calculated using the ImageJ software (ImageJ, U.S. National Institutes of Health, Bethesda, MD, USA).

### 4.9. Quantification of Growth Factors and Bioactive Molecules in Human Sera

The concentrations of the following growth factors and other molecules were measured in blood-derived preparations by using commercially available Quantikine colorimetric sandwich ELISA kits: epidermal growth factor (EGF), fibroblast basic growth factor (FGFb), hepatocyte growth factor (HGF), tumor necrosis factor-alpha (TNFα), and vascular endothelial growth factor (VEGF) (Life Technologies); glial cell line-derived neurotrophic factor (GDNF), insulin-like growth factor-1 (IGF-1), platelet-derived growth factor AB (PDGF-AB), and transforming growth factor-beta 1 (TGF-β1) (RayBiotech^®^, Norcross, Georgia); keratocyte growth factor (KGF or FGF7) (Abnova, Walnut, CA, USA); interferon-gamma (IFNγ) (Diaclone, Besancon Cedex, France); nerve growth factor (NGF) (MyBiosource, San Diego, CA, USA); human substance P (Cusabio, College Park, MD, USA); fibronectin (Biorbyt, Cambridge, UK). Results were expressed as mean ± SD for each human blood-derived preparation.

### 4.10. Statistical Analysis

Each experiment was performed at least three times, and each sample was tested in triplicates. For the statistical analysis, IBM SPSS Statistics 24 (IBM, Armonk, NY, USA) or STATA software (StataCorp LLC, College Station, TX, USA) were used. After running the Shapiro–Wilk test, in case of the normal distribution of the results, one way ANOVA test was used. For the post hoc analysis, Bonferroni was applied in the case of homoscedasticity; on the contrary, Games-Howell non-parametric test was used. In cases of non-parametric data, for the analysis of the variance, the Kruskal–Wallis test was performed. Finally, in order to determine the correlations between the different variables, the non-parametric Spearman test was used.

For further multivariate analysis of data, we performed a principal component analysis (PCA), which is an unsupervised multivariate method that enables variable reduction by building linear combinations called principal component (PC) [85]. The first principal component explains most of the data variance. The second principal component, uncorrelated to the first one, accounts for most of the residual variance and so on. We also carried out cluster analysis, which groups individuals or objects into clusters so that objects in the same cluster are homogeneous and there is heterogeneity across clusters. We combined both techniques, including the factorial scores obtained in the PCA as variables in the hierarchical cluster analysis [86]. Finally, we performed a partial least square discriminant analysis (PLS-DA), a supervised discriminant method based on the PLS regression models, using cross-validation leave-one-out [87]. This analysis requires a priori knowledge about the classes of treatment. PLS-DA was used to extract latent variables of the data set that enable the construction of a factor, allowing predicting a class. We analyzed the predictive capacity for each of the treatments against the rest by means of the area under the ROC curve (AUC). The global prediction capacity of the PLS-DA model has been measured using the “multiclass AUC measure” proposed by Hall and Till [88]. The multivariate statistical techniques were performed using the software R.3.2 (R Core Team, 2013) (R Foundation for Statistical Computing, General Public License, University of Auckland, Auckland, New Zealand).

## 5. Conclusions

In conclusion, the present results support the inter-individual variability that exists when working with biological samples. However, they show that this multivariate statistical analysis can be very informative on the culture factors that specifically influence cell phenotype. Nevertheless, a higher number of biological replicates than the ones we have used in the present study should be required for the results to be conclusive. In addition, our study shows that FBS can be replaced by HS or s-PRGF for the culture of LESCs in explants in order to reduce the use of xenogeneic products. Both HS and s-PRGF help in maintaining the stem/progenitor phenotype in culture. As the growth factors and cytokines are essential for the maintenance of the niche where LESCs are, more studies are required to elucidate what are the bioactive molecules and pathways involved in the self-renewal, quiescence, multipotency, and differentiation status of these cells in order to achieve a complete understanding of their behavior in culture. Furthermore, depending on the type (HS or s-PRGF) and the pool of human sera used for culture, and therefore the growth factors provided by donors as well as the methodology used to produce the serum, different amounts of positive cells for the expression of p63α and other cell markers have been found. Having a small number of biological samples doesn’t allow finding significant and conclusive differences in the behavior of cultures treated with the sera under study. So, further analysis should be done to detect which are the components in serum responsible for the maintenance of the stemness phenotypes of cultures, in order to improve cell therapy for LSCD.

## Figures and Tables

**Table 1:** The concentration of growth factors and other molecules in two different pools of the human blood derivatives—HS and s-PRGF.

**Blood Derivatives**	**Pool**	**EGF** **(pg/mL)**	**HGF** **(pg/mL)**	**FGFb** **(pg/mL)**	**KGF** **(pg/mL)**	**PDGF-AB** **(ng/mL)**	**TGF-β** **(ng/mL)**	**VEGF** **(pg/mL)**
HS	1	277.20 ± 0	59.06 ± 10.76	81.25 ± 0	24.38 ± 0.59	18.89 ± 1.53	5.12 ± 2.34	179.06 ± 5.31
	2	709.76 ± 0	141.11 ± 13.59	123.0 ± 0	56.39 ± 6.92	19.31 ± 2.53	6.83 ± 0.75	280.66 ± 16.42
	Mean ± SD	493.48 ± 305.86	100.08 ± 48.42	109.08 ± 24.1	40.39 ± 18.91	19.10 ± 1.72	5.98 ± 1.73	229.86 ± 59.5
s-PRGF	1	199.24 ± 12.71	28.01 ± 6.95	39.77 ± 0	21.50 ± 0.57	22.81 ± 2.83	5.66 ± 0.80	281.24 ± 233.04
	2	544.48 ± 199.94	60.80 ± 0.04	59.47 ± 0	42.56 ± 6.53	19.53 ± 5.75	9.13 ± 1.08	112.43 ± 17.04
	Mean ± SD	371.85 ± 230.46	44.4 ± 19.35	49.62 ± 13.93	32.03 ± 12.73	21.17 ± 4.16	7.40 ± 2.15	196.83 ± 166.43
**Blood Derivatives**	**Pool**	**FIBRONECTIN** **(µg/mL)**	**IFN-γ** **(pg/mL)**	**TNF-α** **(pg/mL)**	**NGF** **(pg/mL)**	**IGF-1** **(ng/mL)**	**GDNF** **(pg/mL)**	**SP** **(pg/mL)**
HS	1	58.82 ± 11.12	0.00 ± 0	4.11 ± 2.43	60.41 ± 26.88	3.98 ± 0.49	55.21 ± 6.97	20.35 ± 0.31
	2	112.18 ± 2.73	1.06 ± 0.08	10.19 ± 0.84	30.66 ± 7.95	2.47 ± 1.80	22.67 ± 9.07	11.34 ± 1.92
	Mean ± SD	85.5 ± 31.51	0.53 ± 0.61	7.15 ± 3.81	45.53 ± 23.6	3.23 ± 1.39	38.94 ± 19.91 *	15.84 ± 5.32 *
s-PRGF	1	91.72 ± 11.28	0.00 ± 0	10.62 ± 3.31	42.53 ± 10.89	1.38 ± 0.03	0.00 ± 0	3.40 ± 0.39
	2	31.11 ± 2.49	0.95 ± 0.22	11.54 ± 0.02	49.87 ± 31.75	5.44 ± 0	11.98 ± 0	1.25 ± 0.77
	Mean ± SD	61.41 ± 35.62	0.47 ± 0.56	11.08 ± 1.99	46.2 ± 19.84	3.41 ± 2.35	5.99 ± 6.91	2.32 ± 1.34

* Statistically significant differences between human serum (HS) and serum derived from plasma rich in growth factors (s-PRGF) (*p* < 0.05). Epidermal growth factor (EGF); hepatocyte growth factor (HGF); fibroblast basic growth factor (FGFb); keratocyte growth factor (KGF); platelet-derived growth factor AB (PDGF-AB); transforming growth factor-beta 1 (TGF-β1); vascular endothelial growth factor (VEGF); interferon-gamma (IFNγ); tumor necrosis factor-alpha (TNFα); nerve growth factor (NGF); insulin-like growth factor-1 (IGF-1); glial cell line-derived neurotrophic factor (GDNF); and substance P (SP).

**Table 2:** Correlations of variables associated with cells cultured with human sera, between them, and with respect to human sera’s growth factors and molecules.

	<12 μm	12–20 μm	>20 μm	Duplication Time	ICC K14	ICC K12	ICC ΔNp63α	PCR *ABCG2*	PCR *ΔNp63α*	PCR *N-cadherin*	PCR *K14*	PCR *K12*	PCR *Ki67*	EGF	HGF	FGFb	KGF	PDGF-AB	TGF-β	VEGF	Fibronectin	IFN-γ	TNF-α	NGF	IGF-1	GDNF	SP
**<12 μm**																											
**12–20 μm**			92% ***										66% *														
**>20 μm**		92% ***																									
**Duplication Time**												−80% **						−75% *								75% *	
**ICC K14**									63% *					63% *	63% *		63% *			−58% *					58% *		
**ICC K12**									−66% *																		
**ICC ΔNp63α**											−74% **																
**PCR *ABCG2***											60% *																
**PCR *ΔNp63α***					63% *	−66% *																					
**PCR *N-cadherin***																											
**PCR *K14***							−74% **	60% *				62% *															
**PCR *K12***				−80% **							62% *					−65% *		73% **					65% *			−73% **	−65% *
**PCR *Ki67***		66% *																	−67% *								

* Statistically significant correlations (* *p* < 0.05; ** *p* < 0.01; *** *p* < 0.001). ICC: Immunocytochemistry; PCR: polymerase chain reaction.

**Table 3:** Correlations between human sera’s growth factors and molecules.

	EGF	HGF	FGFb	KGF	PDGF-AB	TGF-β	VEGF	Fibronectin	IFN-γ	TNF-α	NGF	IGF-1	GDNF	SP
**EGF**		100% ***	80% **	100% ***		60% *			95% ***					
**HGF**	100% ***		80% **	100% ***		60% *			95% ***					
**FGFb**	80% **	80% **		80% **	−80% **					−60% *			80% **	60% *
**KGF**	100% ***	100% ***	80% **			60% *			95% ***					
**PDGF-AB**			−80% *p * = 0.002							80% **			−100% ***	−80% **
**TGF-β**	60% *	60% *		60% *					0.74% **	80% **				−80% **
**VEGF**								80% **			−60% *	−100% ***		
**Fibronectin**							80% **				−80% **	−80% **		
**IFN-γ**	95% ***	95% ***		95% ***		0.74% ***					−63% *			
**TNF-α**			−60% *		80% **	80% **							−80% **	−100% ***
**NGF**							−60% *	−80% *p * = 0.002	−63% *			60% *		−63% *
**IGF-1**							−100% *	−80% *p * = 0.002			60% *			
**GDNF**			80% **		−100% ***					−80% **				80% **
**SP**			60% *		−80% **	−80% **				−100% ***	−63% *		80% **	

* Statistically significant correlations (* *p* < 0.05; ** *p* < 0.01; *** *p* < 0.001).

**Table 4:** Characteristics of each cluster of the hierarchical 12-cluster analysis. For each cluster, variables of cultured cells (expression of several proteins (ICC) or mRNA (PCR)), as well as the content of the human sera, in growth factors and signaling molecules are shown.

	s-PRGF1	s-PRGF2	HS1	HS2
**Cornea 1**	**Cluster 1**	**Cluster 5**	**Cluster 8**	**Cluster 11**
Variables of cultured cells	− ICC ΔNp63α	+++ ICC ΔNp63α	+++ PCR *Ki67*	− PCR *K14*
	−−− PCR *ABCG2*	++ Size 3	+ PCR *N-Cadherin*, *ΔNp63α*	−− PCR *ABCG2*, *K12*
		−− PCR *N-Cadherin*, Size 2	−−− PCR *K12*	
		−−− PCR *Ki67*		
Content of the sera in GFs	+++ PDGF	+++ TGF	+++ GDNF, SP	+++ HGF, FGFb
	++ VEGF	++ IGF-1	++ NGF	++ KGF, EGF
	− SP	−− FN, VEGF	−−− TNF	−− NGF
	−− EGF, GDNF			
	−−− IGF-1			
**Cornea 2**	**Cluster 2**	**Cluster 6**	**Cluster 9**	**Cluster 12**
Variables of cultured cells	+ PCR *K12*	+++ Size 1	+++ PCR *Ki67*	+++ PCR *K14*, Size 3
	−−− PCR *N-Cadherin*	++ PCR *K14*, *ABCG2*	++ PCR *K14*, *ABCG2*	++ PCR *ABCG2*
		+ PCR *K12*	− Size1	+ PCR *K12*
		−− PCR *Ki67*		−−−Size 2
Content of the sera in GFs	++ PDGF	++ TGF-β, IGF-1	+++ GDNF, SP	+++ HGF, FGFb
	− EGF, GDNF	−− FN, VEGF	++ NGF	++ KGF, EGF
	−− IGF-1		−−− TNF	−− NGF
**Cornea 3**	**Cluster 3**	**Cluster 7**	**Cluster 10**	**Cluster 10**
Variables of cultured cells	+++ PCR *K12*	+++ PCR *N-Cadherin*	− PCR *K12*	− PCR *K12*
	++ ICC ΔNp63α, Size 2	−−− PCR *ΔNp63α*	−−− PCR *K14*, *ΔNp63α*	−−− PCR *K14*, *ΔNp63α*
	− Size 3			
Content of the sera in GFs	++ PDGF	++ TGF-β, IGF-1	+ FGFb	+ FGFb
	− IGF-1	−− FN, VEGF		

Positive (+) and negative (−) statistically significant differences between cluster mean and global mean: + (*p* < 0.05), ++ (*p* < 0.01) and +++ (*p* < 0.001); − (*p* < 0.05), −− (*p* < 0.01) and −−− (*p* < 0.001). Cluster 4 has been excluded from the table because it only contains two samples corresponding to two different treatments. GF: growth factors; Size 1: cell size < 12 µm; Size 2: 12–20 µm; Size 3: cell size > 20 µm.

## Supplementary Materials

Supplementary materials can be found at https://www.mdpi.com/1422-0067/21/17/6132/s1.
