# Supplementary material for: Expansion of Human Limbal Epithelial Stem/Progenitor Cells Using Different Human Sera: A Multivariate Statistical Analysis
Source: Int J Mol Sci. 2020 Aug 25;21(17):6132. doi: 10.3390/ijms21176132 (PMC7503296; doi:10.3390/ijms21176132)
Supplement: Supplementary file 1 [file ijms-21-06132-s001.pdf]

Supplementary Materials

Expansion of Human Limbal Epithelial Stem/Progenitor Cells Using Different Human Sera: A Multivariate Statistical Analysis

Table S1. Numerical data on the variables studied in cell cultures under the following treatments: the gold standard treatment and the explants culture methodology treated with FBS or with pool 1 and pool 2 of the human sera HS and s-PRGF.

		**Treatment**					
		**Gold Std**	**FBS**	**HS1**	**HS2**	**s-PRGF1**	**s-PRGF2**
Cell size (%)	T < 12 μm	6.16 ± 6.29	4.88 ± 1.76	6.38 ± 4.47	1.63 ± 0.94	4.40 ± 1.46	6.29 ± 4.66
Cell Growth	Number of duplications/day	0.54 ± 0.18	0.17 ± 0.06	0.21 ± 0.10	0.30 ± 0.00	0.40 ± 0.04	0.30 ± 0.12
LESC Protein Markers (% of positive cells)	K12	3.00 ± 3.58	3.1 ± 2.6	2.70 ± 2.07	2.00 ± 1.94	1.59 ± 2.68	1.75 ± 2.07
	K14	85.56 ± 4.37	94.06 ± 5.08	93.15 ± 5.32	94.81 ± 5.18	91.32 ± 6.71	95.57 ± 2.99
	P63α	12.29 ± 4.39	10.92 ± 23.93	10.15 ± 7.78	15.34 ± 14.88	13.09 ± 15.44	20.89 ± 21.94
Gene Expression ($2^{(-\Delta\Delta ct)}$)	*ABCG2*	4.75 ± 3.49	0.81 ± 0.29	1.19 ± 0.81	0.80 ± 0.98	0.37 ± 0.30	1.11 ± 0.84
	ΔNP63α	3.43 ± 0.88	1.14 ± 0.50	0.79 ± 0.55	0.85 ± 0.59	0.46 ± 0.26	0.63 ± 0.39
	N-Cadherin	0.67 ± 0.24	0.84 ± 0.15	1.92 ± 1.38	2.23 ± 1.54	1.35 ± 1.08	3.26 ± 4.56
	K14	4.11 ± 3.97	0.61 ± 0.25	0.95 ± 0.83	0.99 ± 1.06	0.86 ± 0.28	1.05 ± 0.82
	K12	18.16 ± 29.78	4.11 ± 1.33	0.09 ± 0.03	0.32 ± 0.43	1.34 ± 1.23	0.53 ± 0.47
	Ki67	3.38 ± 1.04	0.62 ± 0.53	1.90 ± 1.11	0.75 ± 0.14	0.88 ± 0.21	0.46 ± 0.29

Table S2. Confusion table obtained by supervised PLS-DA analysis showing the classification of all samples in the 6 designated treatments, with an overall classification error rate of 24.21%. Based on the data of the designated treatments, the PLS-DA analysis obtains the differentiating functions on the basis of which we can predict which treatment will be a new sample. Comparing the Original (designated) classification of the samples with the Predicted we observe that the cultures under the gold standard and s-PRGF-1 treatments classify perfectly, the prediction being exactly the same as the original classification. For the rest of the treatments, discrimination is worse, since some samples are poorly classified. This is especially evident for HS2, in which no sample corresponding to that treatment is predicted as such, so it has a classification error of 100%.

**Original**	**Predicted**					
	**Gold Std**	**FBS**	**s-PRGF1**	**s-PRGF2**	HS1	HS2
Gold Std	14	0	0	0	0	0
FBS	0	15	0	2	0	0
s-PRGF1	0	0	18	0	0	0
s-PRGF2	0	1	0	10	5	0
HS1	0	0	2	0	15	0
HS2	0	2	2	3	6	0

Table S3. Primers used in RT-qPCR.

Gene	Direction	Primer Sequence (5′→3′)
GAPDH	Forward	CGACCACTTTGTCAAGCTCA
	Reverse	AGGGGTCTACATGGCAACTG
ABCG2	Forward	CCGCGACAGCTTCCAATGACCT
	Reverse	GCCGAAGAGCTGCTGAGAACTGTA
ΔNp63α	Forward	TCCATGGATGATCTGGCAAGT
	Reverse	GCCCTTCCAGATCGCATGT
N-cadherin	Forward	GAGGAGTCAGTGAAGGAGTCA
	Reverse	GGCAAGTTGATTGGAGGGATG
K14	Forward	GACCATTGAGGACCTGAGGA
	Reverse	ATTGATGTCGGCTTCCACAC
K12	Forward	CCAGGTGAGGTCAGCGTAGAA
	Reverse	CCTCCAGGTTGCTGATGAGC
Ki67	Forward	CTTTGGGTGCGACTTGACG
	Reverse	GTCGACCCCGCTCCTTTT

ABCG2, ATP-binding cassette sub-family G member 2; GAPDH, glyceraldehyde-3-phosphate dehydrogenase; K12, cytokeratin 12; K14, cytokeratin 14.

Table S4. Primary antibodies used in immunocytochemistry.

Primary Antibody	Dilution	Source and Catalogue #
K12	1:100	Santa Cruz Biotechnology sc-25722
K14	1:2	Fisher Scientific MS-115-R7
p63*α*	1:100	Cell Signaling Technology #4892

K12, cytokeratin 12; K14, cytokeratin 14. Since the predominant p63 in the limbus is ΔNp63, it is presume that the + cells express ΔNp63.

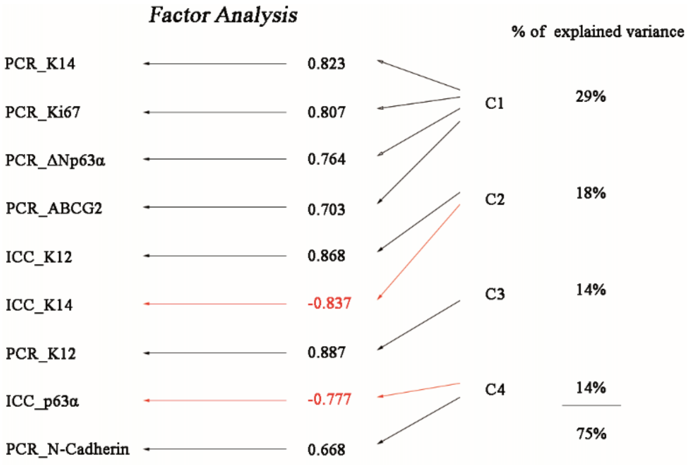

Figure S1. Principal components obtained for the PCA analysis including all treatments. Percentage of explained variance is addressed for each component. Components C1, C2 and C3 explain the same percentage of variance than C1, C2 and C4 (represented in Figure 1), but the latter ones include the variable ICQ_p63*α*, which has been shown to be a very important indicator to discriminate success after LESC transplantation for the treatment of LSCD (Rama et al., 2010).

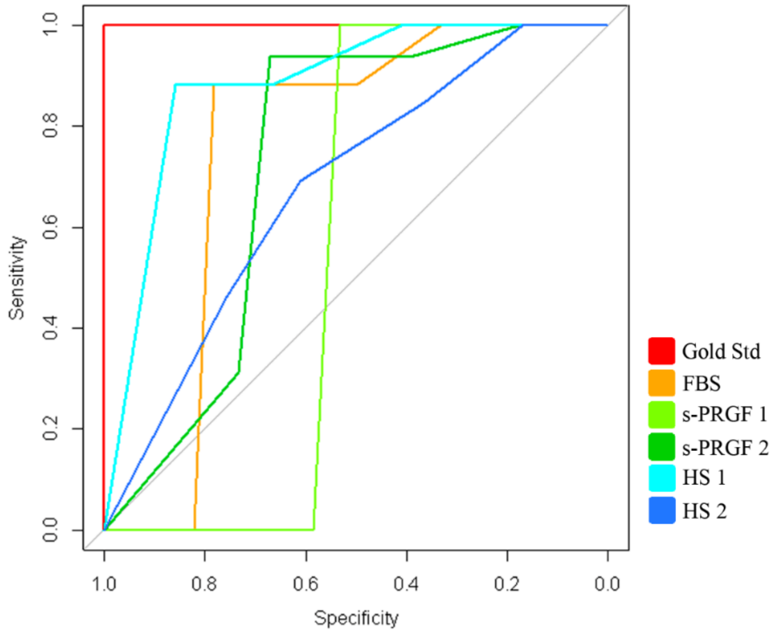

Figure S2. ROC (Receiver Operator Characteristic) curves and AUC (area under the curve) to predict each of the treatments against the rest.

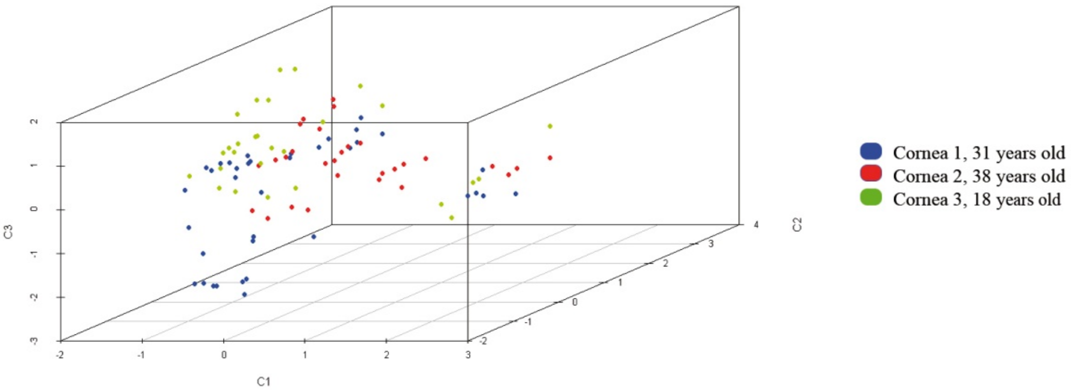

Figure S3. Representation of all samples in three components obtained by PCA analysis. Colors indicate different groups of cornea and amniotic membrane (Cornea-HAM).

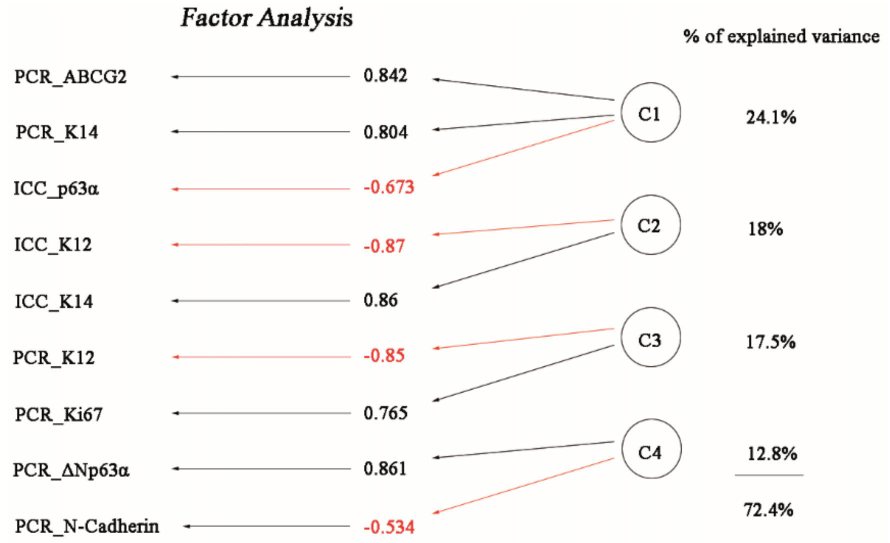

Figure S4. Principal components obtained for the PCA analysis including all treatments except the gold standard treatment. Percentage of explained variance is addressed for each component.

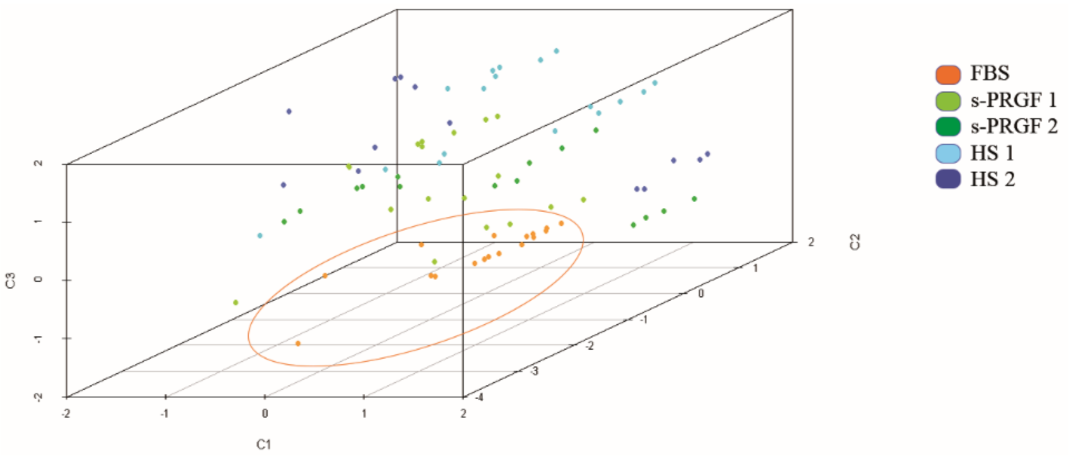

Figure S5. Representation of all samples except the gold standard treatment in the first three components obtained by PCA analysis. Colors indicate treatments. Explants cultures treated with FBS are separated from cultures treated with human sera.

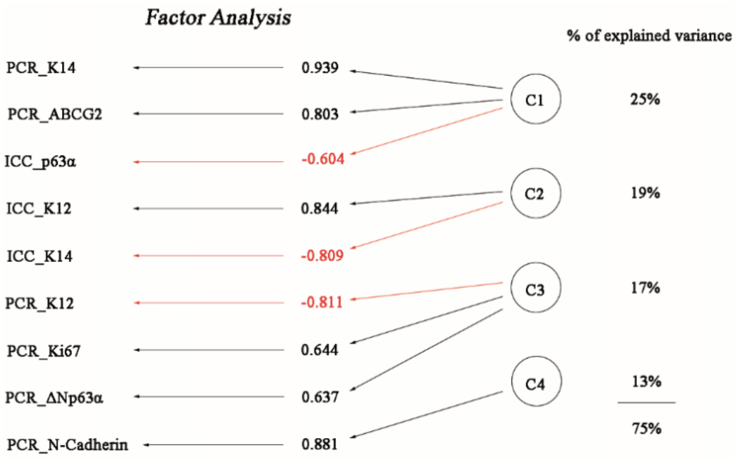

Figure S6. Principal components obtained for the PCA analysis including only cultures treated with human sera. Percentage of explained variance is addressed for each component.

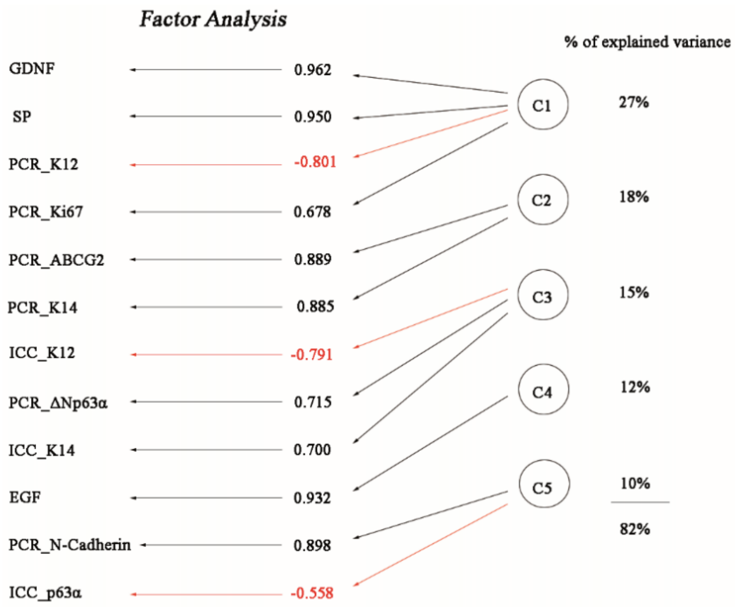

Figure S7. Principal components obtained for the PCA analysis including only cultures treated with human sera, but adding as variables of the analysis some molecules measured in sera, such as GDNF, SP and EGF. Percentage of explained variance is addressed for each component.

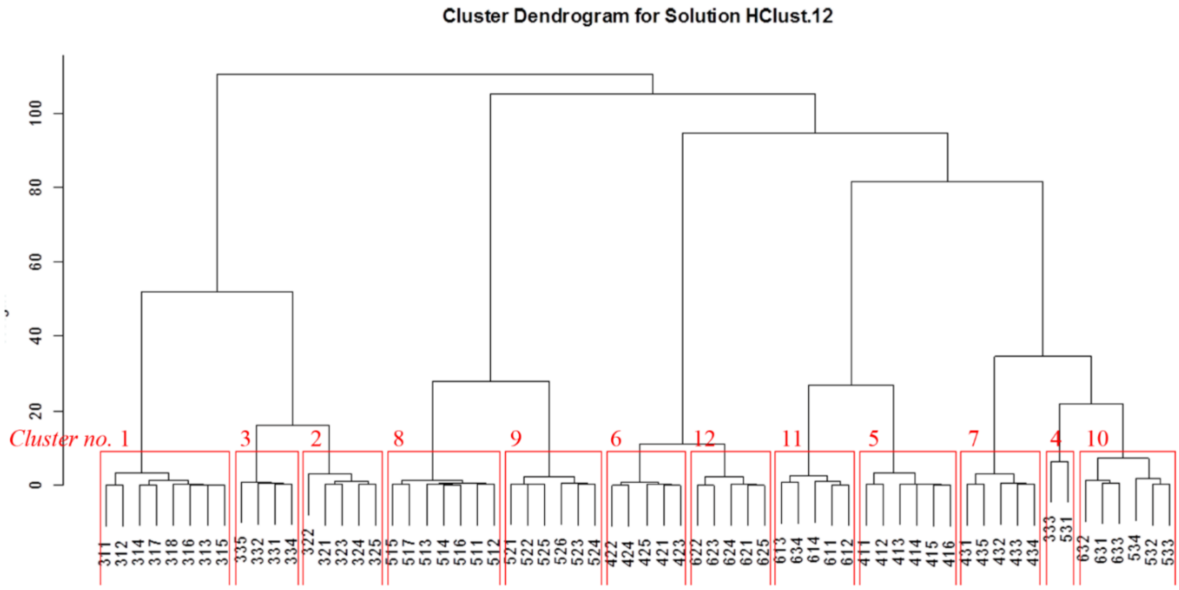

Figure S8. Cluster dendrogram for 12 classes obtained using as variables the five factor scores obtained in the PCA analysis where only samples from cultures treated with human sera and three molecules from sera (GDNF, SP and EGF) were included. Classification provides almost a cluster for each group of a treatment and a Cornea-HAM. For each sample, 1st number means treatment (1: gold std; 2: FBS; 3: s-PRGF1; 4: s-PRGF2; 5: HS1; 6: HS2), 2nd number means the cornea-HAM set (1, 2 or 3), and 3rd number means the number of replicate.
